# Supplementary material for: Uptake of COVID-19 vaccinations amongst 3,433,483 children and young people: meta-analysis of UK prospective cohorts
Source: Nat Commun. 2024 Mar 15;15:2363. doi: 10.1038/s41467-024-46451-0 (PMC10943015; doi:10.1038/s41467-024-46451-0)
Supplement: Supplementary file 1 — Supplementary Information [file 41467_2024_46451_MOESM1_ESM.pdf]

Supplementary material

Contents

Vaccine schedule.......... 2

Results.......... 3

Sensitivity analysis 9

Cohort selection.......... 16

Vaccine schedule

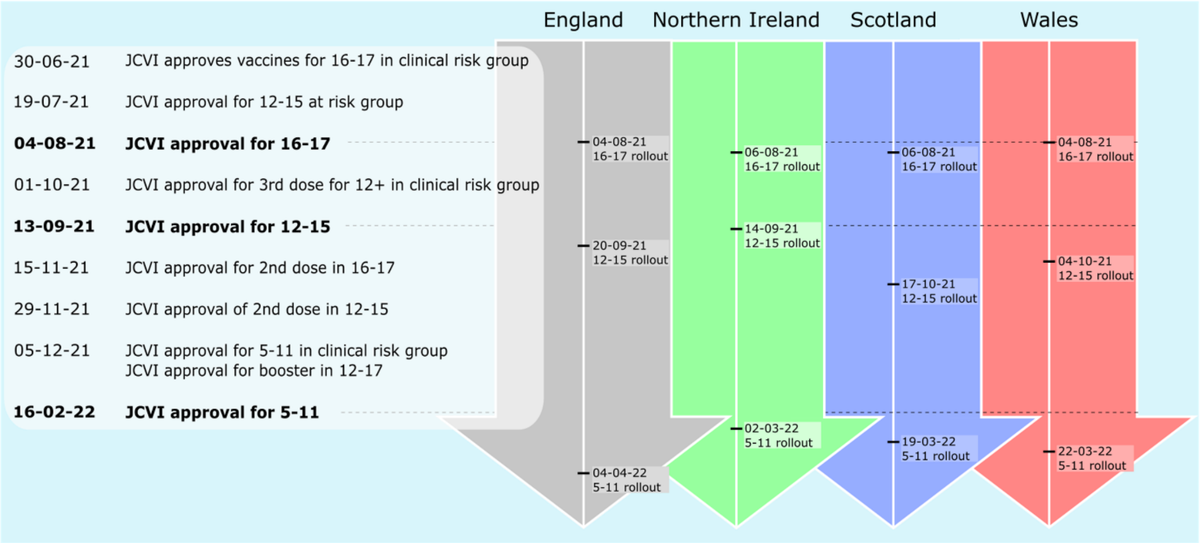

Supplementary Figure 1 The COVID-19 vaccination rollout for CYP in England, Northern Ireland, Scotland and Wales compared to the JCVI approval schedule.

Results

Supplementary Table 1 Cohort breakdown for each variable and the total number of infections by country

		England		Northern Ireland		Scotland		Wales	
		n	%	n	%	n	%	n	%
**Sex**	Male	1,123,212	51	163,342	51	291,267	51	186,970	51
	Female	1,069,729	49	155,095	49	278,145	49	178,280	49
**Age group**	5-11	1,136,520	52	185,591	58	301,774	53	193,920	53
	12-15	728,283	33	92,537	29	182,456	32	126,960	35
	16 - 17	328,138	15	40,309	13	85,182	15	44,370	12
**Number of people in household**	2	145,359	7	16,730	5	60,738	11	19,030	5
	3	437,686	20	55,220	17	144,266	25	66,460	18
	4	763,892	35	101,708	32	205,297	36	131,100	36
	5+	846,004	39	144,779	46	159,111	28	148,650	41
**Household vaccination status**	Fully vaccinated	1,625,171	74	187,804	59	523,333	92	264,470	72
	Partially vaccinated	329,259	15	72,416	23	28,274	5	61,900	17
	Unvaccinated	238,511	11	58,217	18	17,805	3	38,880	11
**Infection**	Individuals who became infected during the study period	307695	14	43838	14	55656	10	44428	12

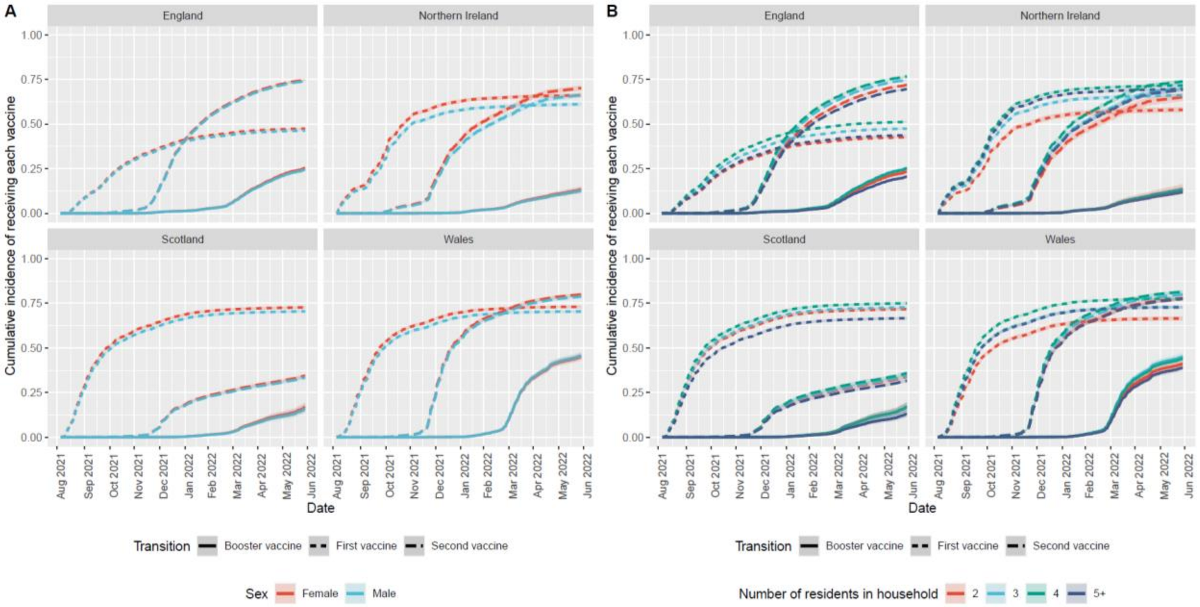

Supplementary Figure 2 Cumulative incidence: The estimated cumulative incidence and 95% confidence intervals for vaccine uptake in each country for each vaccine in a. males vs females and b. household size. All results are relative to the reference group.

*Supplementary Table 2 Multistate model output. Adjusted hazard ratio and 95% confidence intervals for all transitions within each nation for each demographic variable. The reference for each group is aged 16-17, a household occupancy of 3, and a partially vaccinated household. Asterisk indicates significance of two tailed P values, **P<0.05, ***P<0.01.*

Country	*Trans-ition*	*Male*	*Age 12-15*	*Age 5-11*	*Household n = 2*	*Household n = 4*	*Household n = 5*	*Unvaccinated*	*Fully vaccinated*
England	1	0.93 (0.92, 0.94)***	1.32 (1.3, 1.33)***	0.26 (0.25, 0.26)***	0.9 (0.88, 0.92)***	1.09 (1.08, 1.1)***	0.88 (0.87, 0.89)***	0.67 (0.65, 0.68)***	1.56 (1.54, 1.58)***
	2	0.97 (0.96, 0.97)***	0.72 (0.72, 0.72)***	0.11 (0.11, 0.11)***	0.86 (0.85, 0.87)***	1.11 (1.11, 1.12)***	0.89 (0.89, 0.9)***	0.11 (0.11, 0.11)***	2.83 (2.8, 2.85)***
	3	1.51 (0.83, 2.76)	0.24 (0.11, 0.51)***	0.36 (0.16, 0.86)**	1.71 (0.61, 4.84)	0.92 (0.39, 2.15)	0.93 (0.41, 2.14)	0.95 (0.31, 2.93)	1.54 (0.65, 3.66)
	4	1.01 (1, 1.01)**	1.25 (1.23, 1.26)***	2.17 (2.14, 2.19)***	1.02 (1, 1.03)**	0.99 (0.98, 1)**	1.04 (1.03, 1.05)***	1.11 (1.09, 1.13)***	0.87 (0.86, 0.88)***
	5	0.98 (0.96, 0.99)**	0.98 (0.96, 0.99)***	0.11 (0.1, 0.13)***	0.89 (0.86, 0.92)***	1.09 (1.07, 1.11)***	0.91 (0.89, 0.93)***	0.11 (0.1, 0.13)***	2.29 (2.22, 2.37)***
	6	0.88 (0.84, 0.93)***	0.16 (0.15, 0.17)***	0.71 (0.09, 5.47)	0.82 (0.74, 0.91)***	1.09 (1.02, 1.15)**	0.79 (0.74, 0.85)***	0.11 (0.07, 0.18)***	3.35 (3.01, 3.73)***
	7	1.01 (0.06, 16.21)	2724.73 (0, Inf)	1.01 (0, Inf)	0.96 (0, Inf)	0.97 (0, Inf)	13389.32 (0, Inf)	3.46 (0, Inf)	6203.43 (0, Inf)
	8	0.92 (0.91, 0.94)***	1.18 (1.16, 1.2)***	0.11 (0.1, 0.12)***	0.91 (0.88, 0.94)***	1.1 (1.08, 1.12)***	0.9 (0.88, 0.92)***	0.64 (0.56, 0.72)***	1.37 (1.32, 1.41)***
	9	0.98 (0.98, 0.99)***	0.84 (0.84, 0.85)***	0.8 (0.77, 0.84)***	0.92 (0.91, 0.93)***	1.05 (1.04, 1.06)***	0.86 (0.86, 0.87)***	0.59 (0.56, 0.61)***	1.3 (1.29, 1.32)***
	10	1.1 (0.42, 2.85)	0.4 (0.14, 1.16)*	0.71 (0.08, 6.52)	2.26 (0.37, 13.6)	1.29 (0.33, 4.99)	0.72 (0.16, 3.23)	0 (0, Inf)	0.3 (0.09, 1.03)*
	11	0.74 (0.7, 0.77)***	0.09 (0.09, 0.1)***	0 (0, Inf)	0.88 (0.79, 0.98)**	1.03 (0.97, 1.1)	0.81 (0.76, 0.87)***	0.89 (0.57, 1.4)	1.34 (1.2, 1.49)***
	12	0.97 (0.95, 0.98)***	0.06 (0.06, 0.07)***	0 (0, Inf)	0.91 (0.88, 0.95)***	0.99 (0.97, 1.01)	0.79 (0.77, 0.81)***	0.62 (0.51, 0.74)***	1.31 (1.26, 1.36)***
	13	3.3 (0.91, 11.98)*	0.6 (0.17, 2.07)	NA (NA, NA)	0.88 (0.1, 7.95)	0.4 (0.09, 1.8)	0.64 (0.16, 2.63)	0 (0, Inf)	0.28 (0.07, 1.14)*
	14	NA (NA, NA)	NA (NA, NA)	NA (NA, NA)	NA (NA, NA)	NA (NA, NA)	NA (NA, NA)	NA (NA, NA)	NA (NA, NA)
Northern Ireland	1	0.88 (0.86, 0.9)***	0.99 (0.97, 1.02)	0.25 (0.25, 0.26)***	0.89 (0.84, 0.94)***	1.1 (1.06, 1.13)***	1.03 (1, 1.07)**	0.81 (0.78, 0.84)***	1.35 (1.32, 1.38)***
	2	0.87 (0.86, 0.88)***	0.45 (0.44, 0.45)***	0 (0, 0)***	0.8 (0.77, 0.83)***	1.17 (1.14, 1.19)***	1.1 (1.08, 1.13)***	0.24 (0.23, 0.25)***	2.33 (2.29, 2.37)***
	3	0 (0, Inf)	0.6 (0, Inf)	2348.43 (0, Inf)	0.99 (0, Inf)	1.05 (0, Inf)	23616.48 (0, Inf)	2.76 (0, Inf)	18393.09 (0, Inf)
	4	1.02 (1, 1.04)**	1.25 (1.22, 1.28)***	2.11 (2.05, 2.17)***	1.02 (0.97, 1.06)	0.98 (0.96, 1)	1.01 (0.98, 1.03)	1.07 (1.04, 1.1)***	0.88 (0.87, 0.9)***
	5	0.9 (0.86, 0.95)***	0.62 (0.59, 0.65)***	0 (0, 0.01)***	0.81 (0.7, 0.94)***	1.19 (1.11, 1.28)***	1.14 (1.06, 1.22)***	0.18 (0.14, 0.21)***	2.49 (2.33, 2.66)***
	6	0.67 (0.57, 0.78)***	0.06 (0.04, 0.08)***	0 (0, Inf)	0.48 (0.29, 0.8)**	1.26 (1.01, 1.58)**	1.31 (1.05, 1.63)**	0.05 (0.02, 0.15)***	4.22 (3.38, 5.26)***
	7	NA (NA, NA)	NA (NA, NA)	NA (NA, NA)	NA (NA, NA)	NA (NA, NA)	NA (NA, NA)	NA (NA, NA)	NA (NA, NA)
	8	0.83 (0.79, 0.87)***	1.19 (1.13, 1.26)***	0.18 (0.06, 0.56)***	0.79 (0.68, 0.91)***	1.13 (1.05, 1.21)***	1.08 (1.01, 1.16)**	0.67 (0.56, 0.81)***	1.59 (1.49, 1.7)***
	9	0.9 (0.88, 0.92)***	0.3 (0.29, 0.31)***	1.36 (0.96, 1.92)*	0.87 (0.82, 0.92)***	1.11 (1.07, 1.14)***	0.97 (0.94, 1.01)	0.57 (0.53, 0.61)***	1.5 (1.46, 1.54)***
	10	NA (NA, NA)	NA (NA, NA)	NA (NA, NA)	NA (NA, NA)	NA (NA, NA)	NA (NA, NA)	NA (NA, NA)	NA (NA, NA)
	11	0.57 (0.49, 0.67)***	0.15 (0.11, 0.2)***	0 (0, Inf)	0.58 (0.35, 0.97)**	0.93 (0.75, 1.17)	1.05 (0.85, 1.31)	0.32 (0.1, 1.02)*	1.61 (1.3, 2.01)***
	12	0.95 (0.88, 1.02)	0.16 (0.14, 0.18)***	0 (0, Inf)	1.03 (0.84, 1.25)	0.99 (0.89, 1.11)	0.89 (0.8, 0.99)**	0.62 (0.44, 0.88)**	1.37 (1.24, 1.52)***
	13	NA (NA, NA)	NA (NA, NA)	NA (NA, NA)	NA (NA, NA)	NA (NA, NA)	NA (NA, NA)	NA (NA, NA)	NA (NA, NA)
	14	NA (NA, NA)	NA (NA, NA)	NA (NA, NA)	NA (NA, NA)	NA (NA, NA)	NA (NA, NA)	NA (NA, NA)	NA (NA, NA)
Scotland	1	0.93 (0.91, 0.95)***	0.84 (0.82, 0.86)***	0.25 (0.24, 0.26)***	1 (0.96, 1.03)	1 (0.97, 1.03)	0.87 (0.85, 0.89)***	0.86 (0.81, 0.92)***	1.08 (1.03, 1.12)***
	2	0.94 (0.93, 0.95)***	0.77 (0.77, 0.78)***	0.17 (0.17, 0.17)***	0.97 (0.96, 0.99)***	1.07 (1.06, 1.08)***	0.85 (0.84, 0.85)***	0.29 (0.27, 0.3)***	1.58 (1.55, 1.61)***
	3	1.79 (0.61, 5.24)	0.59 (0.19, 1.83)	0.18 (0.03, 1.15)*	0.45 (0.05, 3.89)	0.74 (0.21, 2.55)	0.54 (0.14, 2.03)	0.63 (0.06, 7.16)	0.54 (0.12, 2.5)
	4	1.02 (1.01, 1.04)***	1.82 (1.78, 1.86)***	1.82 (1.77, 1.88)***	1.01 (0.98, 1.04)	0.99 (0.96, 1.01)	1.03 (1.01, 1.06)**	1.02 (0.96, 1.08)	0.81 (0.78, 0.84)***
	5	0.93 (0.9, 0.96)***	0.71 (0.68, 0.73)***	0.01 (0.01, 0.02)***	0.97 (0.92, 1.03)	1.09 (1.05, 1.14)***	0.91 (0.87, 0.95)***	0.25 (0.18, 0.35)***	3.02 (2.69, 3.41)***
	6	0.84 (0.74, 0.96)**	0.08 (0.06, 0.1)***	NA (NA, NA)	1.11 (0.89, 1.37)	1.13 (0.96, 1.34)	0.77 (0.64, 0.94)**	0.34 (0.07, 1.56)	6.59 (3.53, 12.3)***
	7	3749.63 (0, Inf)	1.33 (0, Inf)	6312.12 (0, Inf)	1906506.46 (0, Inf)	0.96 (0, Inf)	1.17 (0, Inf)	0 (0, Inf)	0.87 (0, Inf)
	8	0.86 (0.84, 0.89)***	0.63 (0.61, 0.65)***	0.01 (0, 0.01)***	0.96 (0.9, 1.02)	1.08 (1.04, 1.13)***	0.95 (0.9, 0.99)**	0.88 (0.63, 1.24)	3.48 (3.09, 3.92)***
	9	0.97	1.1	0.47	0.98	1.05	0.9	0.79	3.79

		(0.96, 0.98)***	(1.08, 1.11)***	(0.44, 0.5)***	(0.96, 1)**	(1.04, 1.07)***	(0.89, 0.92)***	(0.71, 0.87)***	(3.66, 3.93)***
	10	1.19	0.38	0	6.69	0.72	3.23	0	0.22
		(0.32, 4.45)	(0.09, 1.62)	(0, Inf)	(0.7, 64.45)	(0.04, 11.51)	(0.36, 29.26)	(0, Inf)	(0.05, 0.97)*
	11	0.72	0.03	0	1.13	0.99	0.9	2.04	1.6
		(0.63, 0.83)***	(0.02, 0.03)***	(0, Inf)	(0.91, 1.4)	(0.84, 1.17)	(0.74, 1.09)	(0.45, 9.34)	(0.86, 2.99)
	12	0.94	0.07	0	1.02	1.01	0.77	0.96	1.79
		(0.9, 0.97)***	(0.06, 0.07)***	(0, Inf)	(0.96, 1.08)	(0.97, 1.06)	(0.73, 0.81)***	(0.57, 1.6)	(1.53, 2.09)***
	13	0.98	1.91	NA	0	1.32	3.23	2.02	1843.47
		(0.2, 4.87)	(0.22, 16.53)	(NA, NA)	(0, Inf)	(0.12, 14.58)	(0.34, 31.05)	(0, Inf)	(0, Inf)
	14	NA	NA	NA	NA	NA	NA	NA	NA
		(NA, NA)	(NA, NA)		(NA, NA)	(NA, NA)	(NA, NA)	(NA, NA)	(NA, NA)
Wales	1	0.92	1.58	0.1	0.96	1.11	1.02	0.78	1.3
		(0.9, 0.94)***	(1.53, 1.64)***	(0.09, 0.1)***	(0.91, 1.02)	(1.07, 1.14)***	(0.98, 1.05)	(0.74, 0.81)***	(1.26, 1.34)***
	2	0.93	0.46	0.06	0.83	1.14	0.99	0.19	2.45
		(0.92, 0.94)***	(0.45, 0.46)***	(0.06, 0.06)***	(0.81, 0.86)***	(1.12, 1.16)***	(0.98, 1.01)	(0.18, 0.2)***	(2.41, 2.49)***
	3	0.64	0.41	0.16	1.54	0.55	1.61	0.74	2.03
		(0.2, 2.03)	(0.1, 1.67)	(0.03, 0.9)**	(0.14, 17.1)	(0.08, 3.88)	(0.33, 7.96)	(0.07, 8.47)	(0.41, 9.98)
	4	1.02	1.91	3.45	1.05	0.99	1.02	1.09	0.87
		(1, 1.04)**	(1.85, 1.97)***	(3.31, 3.59)***	(1, 1.1)*	(0.96, 1.02)	(0.99, 1.05)	(1.04, 1.13)***	(0.85, 0.89)***
	5	0.96	0.6	0.05	0.86	1.06	0.94	0.22	1.97
		(0.92, 1)**	(0.58, 0.62)***	(0.04, 0.07)***	(0.77, 0.95)***	(1, 1.11)**	(0.89, 0.99)**	(0.18, 0.27)***	(1.85, 2.11)***
	6	0.88	0.33	0	0.96	1.28	1.01	0.06	2.81
		(0.78, 0.99)**	(0.28, 0.38)***	(0, Inf)	(0.72, 1.29)	(1.08, 1.51)***	(0.84, 1.2)	(0.02, 0.2)***	(2.25, 3.51)***
	7	NA	NA	NA	NA	NA	NA	NA	NA
		(NA, NA)	(NA, NA)	(NA, NA)	(NA, NA)	(NA, NA)	(NA, NA)	(NA, NA)	(NA, NA)
	8	0.87	0.92	0.03	0.87	1.15	1.03	0.83	1.34
		(0.84, 0.9)***	(0.88, 0.96)***	(0.02, 0.04)***	(0.78, 0.96)**	(1.09, 1.21)***	(0.98, 1.09)	(0.68, 1.02)*	(1.26, 1.43)***
	9	0.97	0.96	0.42	0.94	1.05	0.93	0.64	1.3
		(0.95, 0.98)***	(0.95, 0.97)***	(0.38, 0.47)***	(0.91, 0.97)***	(1.03, 1.07)***	(0.91, 0.95)***	(0.6, 0.68)***	(1.28, 1.33)***
	10	1.24	0.71	0	0	2.14	1.12	1.14	4517.74
		(0.28, 5.56)	(0.13, 3.92)	(0, Inf)	(0, Inf)	(0.24, 19.16)	(0.1, 12.4)	(0, Inf)	(0, Inf)
	11	0.66	0.1	0	1.2	1.32	1.14	0.32	1.45
		(0.58, 0.74)***	(0.09, 0.12)***	(0, Inf)	(0.9, 1.61)	(1.12, 1.56)***	(0.96, 1.36)	(0.1, 1.02)*	(1.16, 1.81)***
	12	1.02	0.14	NA	0.89	0.97	0.82	0.5	1.38
		(0.99, 1.05)	(0.14, 0.15)***	(NA, NA)	(0.83, 0.95)***	(0.93, 1.01)	(0.79, 0.86)***	(0.41, 0.6)***	(1.31, 1.45)***
	13	2.06	0.23	NA	0	0.21	0	0	0.12
		(0.19, 22.77)	(0.02, 2.62)	(NA, NA)	(0, Inf)	(0.02, 2.35)	(0, Inf)	(0, Inf)	(0.01, 1.38)*
	14	NA	NA	NA	NA	NA	NA	NA	NA
		(NA, NA)	(NA, NA)	(NA, NA)	(NA, NA)	(NA, NA)	(NA, NA)	(NA, NA)	(NA, NA)

Supplementary Table 3 Adjusted and Unadjusted hazard ratios from the multistate model for Wales

Variable	Type	Transition	Adjusted Hazard Ratio	Unadjusted Hazard Ratio
sex	Male	2	0.93 (0.92, 0.94)	0.96 (0.95, 0.97)
		9	0.97 (0.95, 0.98)	0.97 (0.96, 0.98)
		12	1.02 (0.99, 1.05)	1.01 (0.98, 1.04)
age category	12-15	2	0.46 (0.45, 0.46)	0.5 (0.5, 0.51)
		9	0.96 (0.95, 0.97)	0.96 (0.94, 0.97)
		12	0.14 (0.14, 0.15)	0.15 (0.15, 0.16)
	5-11	2	0.06 (0.06, 0.06)	0.07 (0.07, 0.07)
		9	0.42 (0.38, 0.47)	0.35 (0.31, 0.4)
		12	NA	NA
household n	2	2	0.83 (0.81, 0.86)	0.87 (0.85, 0.9)
		9	0.94 (0.91, 0.97)	0.95 (0.92, 0.98)
		12	0.89 (0.83, 0.95)	0.93 (0.87, 1)
	4	2	1.14 (1.12, 1.16)	1.13 (1.12, 1.15)
		9	1.05 (1.03, 1.07)	1.05 (1.03, 1.07)
		12	0.97 (0.93, 1.01)	0.91 (0.88, 0.95)
	5	2	0.99 (0.98, 1.01)	0.89 (0.88, 0.9)
		9	0.93 (0.91, 0.95)	0.89 (0.88, 0.91)
		12	0.82 (0.79, 0.86)	0.75 (0.72, 0.78)
vaccinated household	Unvaccinated	2	0.19 (0.18, 0.2)	0.18 (0.17, 0.19)
		9	0.64 (0.6, 0.68)	0.66 (0.62, 0.7)
		12	0.5 (0.41, 0.6)	0.59 (0.48, 0.71)
	Fully vaccinated	2	2.45 (2.41, 2.49)	2.14 (2.1, 2.18)
		9	1.3 (1.28, 1.33)	1.34 (1.31, 1.37)
		12	1.38 (1.31, 1.45)	1.34 (1.27, 1.4)

Supplementary Table 4 Results of the meta-analysis before removal of Northern Irelands 5-11 year olds. Please note the very large τ² resulting from small numbers in Northern Irelands 5-11-year-olds receiving their vaccine.

Variable	*Type*	*Transition*	*aHR*	*95% CI lower*	*95% CI upper*	*Between study variance (τ²)*
**Age**	*5-11*	1st dose	0.03	0.00	0.31	5.00
		2nd dose	0.67	0.4 0	1.12	0.26
	12-15	1st dose	0.58	0.43	0.77	0.09
		2nd dose	0.72	0.40	1.28	0.35
		Booster dose	0.10	0.06	0.16	0.23
**Sex**	*Male*	1st dose	0.93	0.89	0.97	0.00
		2nd dose	0.95	0.92	0.99	0.00
		Booster dose	0.97	0.93	1.01	0.00
**Number of residents in household**	*2*	1st dose	0.87	0.80	0.94	0.01
		2nd dose	0.93	0.89	0.97	0.00
		Booster dose	0.95	0.88	1.02	0.00
	4	1st dose	1.12	1.08	1.16	0.00
		2nd dose	1.06	1.04	1.08	0.00
		Booster dose	0.99	0.97	1.01	0.00
	5+	1st dose	0.95	0.85	1.07	0.01
		2nd dose	0.92	0.87	0.96	0.00
		Booster dose	0.80	0.77	0.84	0.00
**Vaccination status of adults in household**	*Unvaccinated*	1st dose	0.19	0.13	0.29	0.18
		2nd dose	0.64	0.56	0.73	0.02
		Booster dose	0.60	0.50	0.73	0.02
	Fully vaccinated	1st dose	2.25	1.76	2.87	0.06
		2nd dose	1.76	1.06	2.92	0.26
		Booster dose	1.43	1.27	1.61	0.01

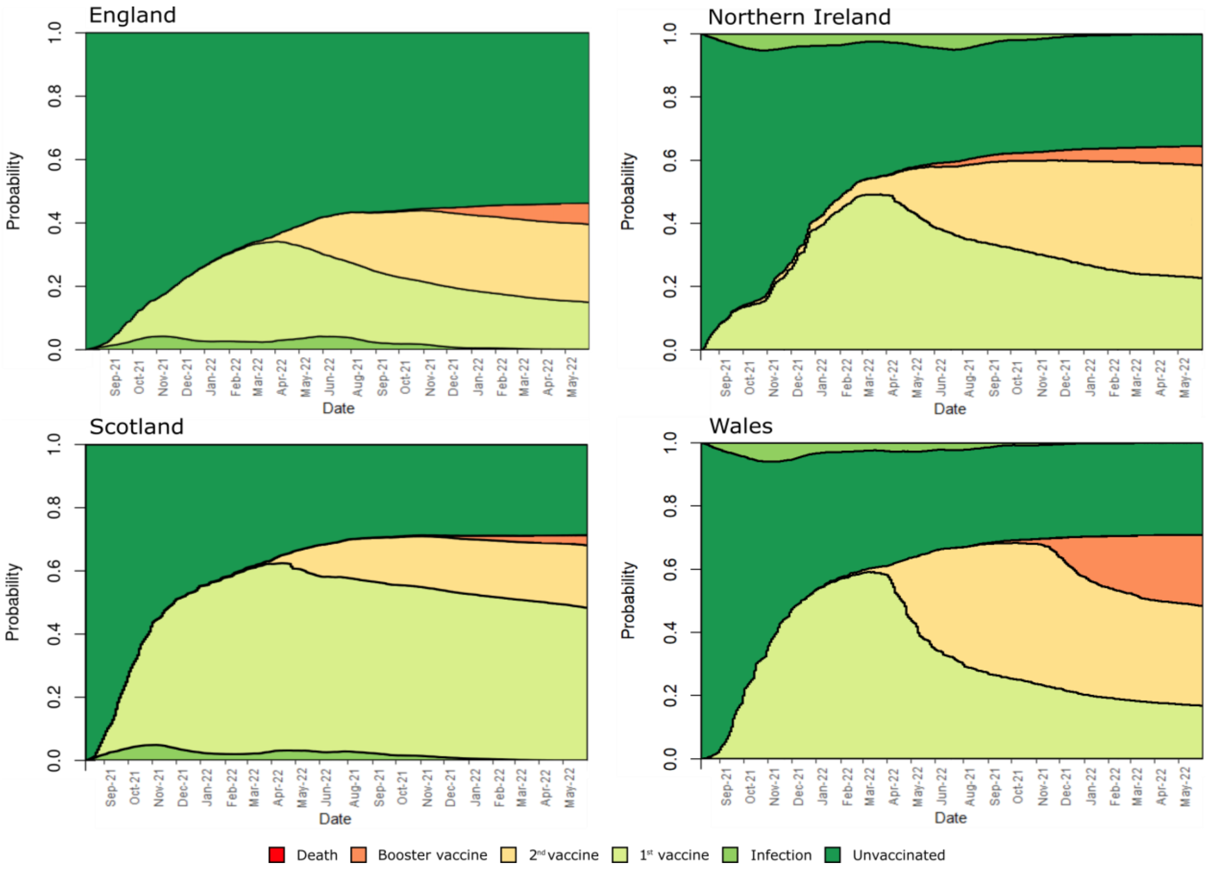

Supplementary Figure 3 Cumulative probability plots representing the different states of the mode, and the probability that an individual will occupy each state throughout the study period.

Sensitivity analysis

We conducted a sensitivity analysis to investigate the effect of expanding the study period until the end of 2022 (31st Dec), and to account for additional covariates.

England were unable to contribute data for the additional covariates and extended study period. Northern Ireland were unable to provide ethnicity data due to small numbers.

*Supplementary Table 5 Adjusted hazard ratio and 95% confidence intervals resulting from the multistate model vaccination transitions for Northern Ireland, Scotland and Wales with a study end date of 31st May 2022. Northern Ireland did not provide results for the ethnicity. The reference for each group is aged 16-17, female, a household occupancy of three, a partially vaccinated household, urban, IMD 2019 quintile of three and white. Asterisk indicates significance of two tailed P values, **P<0.05, ***P<0.01.*

		Sex	Age group		Household n =			Household vaccination status	
Country	Vaccine	Male	12-15	5-11	2	4	5+	Unvaccinated	Fully vaccinated
Wales	1st dose	0.93 (0.92, 0.94) (P = 0 ***)	0.44 (0.44, 0.45) (P = 0 ***)	0.06 (0.06, 0.06) (P = 0 ***)	0.86 (0.83, 0.88) (P = 0 ***)	1.12 (1.11, 1.14) (P = 0 ***)	1.01 (1, 1.03) (P = 0.1)	0.2 (0.19, 0.21) (P = 0 ***)	2.43 (2.39, 2.47) (P = 0 ***)
	2nd dose	0.97 (0.95, 0.98) (P = 0 ***)	0.94 (0.93, 0.95) (P = 0 ***)	0.45 (0.45, 0.46) (P = 0 ***)	0.96 (0.93, 0.99) (P = 0.01 **)	1.04 (1.03, 1.06) (P = 0 ***)	0.93 (0.91, 0.94) (P = 0 ***)	0.66 (0.62, 0.7) (P = 0 ***)	1.28 (1.26, 1.31) (P = 0 ***)
	3rd dose	0.98 (0.96, 1) (P = 0.1)	0.17 (0.16, 0.17) (P = 0 ***)	0 (0, 0.01) (P = 0 ***)	0.92 (0.86, 0.97) (P = 0 ***)	0.97 (0.94, 1) (P = 0.04 **)	0.83 (0.8, 0.86) (P = 0 ***)	0.51 (0.43, 0.6) (P = 0 ***)	1.34 (1.28, 1.39) (P = 0 ***)
Scotland	1st dose	0.94 (0.93, 0.94) (P = 0 ***)	0.57 (0.57, 0.58) (P = 0 ***)	0.12 (0.12, 0.12) (P = 0 ***)	1 (0.99, 1.02) (P = 0.65)	1.03 (1.02, 1.04) (P = 0 ***)	0.84 (0.83, 0.85) (P = 0 ***)	0.31 (0.29, 0.32) (P = 0 ***)	1.44 (1.42, 1.47) (P = 0 ***)
	2nd dose	0.97 (0.96, 0.98) (P = 0 ***)	0.9 (0.88, 0.91) (P = 0 ***)	0.41 (0.39, 0.44) (P = 0 ***)	0.99 (0.97, 1.01) (P = 0.15)	1.03 (1.02, 1.05) (P = 0 ***)	0.93 (0.92, 0.95) (P = 0 ***)	0.81 (0.73, 0.9) (P = 0 ***)	4.6 (4.45, 4.76) (P = 0 ***)
	3rd dose	0.89 (0.85, 0.93) (P = 0 ***)	0.07 (0.06, 0.07) (P = 0 ***)	NA (NA, NA) (P = NA)	1.06 (0.99, 1.14) (P = 0.1)	0.99 (0.94, 1.05) (P = 0.85)	0.78 (0.73, 0.83) (P = 0 ***)	0.68 (0.17, 2.76) (P = 0.59)	1.52 (1.3, 1.79) (P = 0 ***)
Northern Ireland	1st dose	0.88 (0.87, 0.89) (P = 0 ***)	0.44 (0.43, 0.44) (P = 0 ***)		0.82 (0.79, 0.85) (P = 0 ***)	1.14 (1.12, 1.17) (P = 0 ***)	1.1 (1.08, 1.12) (P = 0 ***)	0.24 (0.23, 0.25) (P = 0 ***)	2.27 (2.23, 2.31) (P = 0 ***)
	2nd dose	0.92 (0.91, 0.94) (P = 0 ***)	0.39 (0.38, 0.4) (P = 0 ***)		0.93 (0.88, 0.98) (P = 0.01 **)	1.05 (1.02, 1.09) (P = 0 ***)	0.97 (0.94, 0.99) (P = 0.01 **)	0.59 (0.55, 0.64) (P = 0 ***)	1.38 (1.35, 1.42) (P = 0 ***)
	3rd dose	1.01 (0.96, 1.06) (P = 0.81)	0.48 (0.45, 0.51) (P = 0 ***)		0.98 (0.86, 1.13) (P = 0.83)	1 (0.93, 1.08) (P = 0.98)	0.95 (0.88, 1.02) (P = 0.17)	0.54 (0.4, 0.71) (P = 0 ***)	1.32 (1.23, 1.42) (P = 0 ***)

		Urbanicity	IMD 2019 quintile				Ethnicity				
Country	Vaccine	Rural	1 Most	2	4	5 Least	Asian	Mixed	Other	Black	Unknown
Wales	1st dose	0.96 (0.95, 0.97) (P = 0 ***)	0.83 (0.81, 0.84) (P = 0 ***)	0.95 (0.94, 0.97) (P = 0 ***)	1.09 (1.07, 1.11) (P = 0 ***)	1.28 (1.26, 1.31) (P = 0 ***)	1.3 (1.26, 1.33) (P = 0 ***)	0.88 (0.85, 0.91) (P = 0 ***)	0.91 (0.86, 0.96) (P = 0 ***)	0.83 (0.78, 0.88) (P = 0 ***)	0.35 (0.31, 0.4) (P = 0 ***)
	2nd dose	0.93 (0.91, 0.94) (P = 0 ***)	0.91 (0.9, 0.93) (P = 0 ***)	1 (0.98, 1.02) (P = 0.85)	1.05 (1.03, 1.07) (P = 0 ***)	1.17 (1.14, 1.19) (P = 0 ***)	1.16 (1.12, 1.19) (P = 0 ***)	1 (0.96, 1.04) (P = 0.89)	1.03 (0.97, 1.09) (P = 0.4)	0.96 (0.89, 1.03) (P = 0.22)	0.77 (0.66, 0.91) (P = 0 ***)
	3rd dose	0.85 (0.83, 0.88) (P = 0 ***)	0.88 (0.84, 0.91) (P = 0 ***)	0.95 (0.91, 0.99) (P = 0.01 **)	1.01 (0.97, 1.06) (P = 0.48)	1.23 (1.18, 1.27) (P = 0 ***)	1.04 (0.98, 1.11) (P = 0.19)	1.02 (0.93, 1.11) (P = 0.69)	0.81 (0.71, 0.93) (P = 0 ***)	0.75 (0.62, 0.89) (P = 0 ***)	1.3 (0.89, 1.92) (P = 0.18)
Scotland	1st dose	0.97 (0.96, 0.98) (P = 0 ***)	0.74 (0.72, 0.75) (P = 0 ***)	0.86 (0.85, 0.87) (P = 0 ***)	1.11 (1.09, 1.12) (P = 0 ***)	1.29 (1.27, 1.3) (P = 0 ***)	1.27 (1.25, 1.3) (P = 0 ***)	0.9 (0.87, 0.93) (P = 0 ***)	0.9 (0.85, 0.95) (P = 0 ***)	0.83 (0.79, 0.86) (P = 0 ***)	0.93 (0.93, 0.94) (P = 0 ***)
	2nd dose	0.97 (0.96, 0.99) (P = 0 ***)	0.85 (0.84, 0.87) (P = 0 ***)	0.91 (0.89, 0.93) (P = 0 ***)	1.09 (1.07, 1.11) (P = 0 ***)	1.19 (1.17, 1.21) (P = 0 ***)	0.99 (0.96, 1.02) (P = 0.39)	0.89 (0.84, 0.93) (P = 0 ***)	0.81 (0.75, 0.88) (P = 0 ***)	0.82 (0.77, 0.88) (P = 0 ***)	1.02 (1.01, 1.03) (P = 0 ***)
	3rd dose	1.01 (0.95, 1.06) (P = 0.82)	0.78 (0.72, 0.85) (P = 0 ***)	0.85 (0.79, 0.92) (P = 0 ***)	1.14 (1.07, 1.22) (P = 0 ***)	1.28 (1.2, 1.36) (P = 0 ***)	0.85 (0.74, 0.97) (P = 0.01 **)	1 (0.82, 1.22) (P = 0.97)	0.83 (0.58, 1.2) (P = 0.32)	0.61 (0.41, 0.91) (P = 0.02 **)	0.96 (0.91, 1.01) (P = 0.09 *)
Northern Ireland	1st dose	1.02 (1.01, 1.04) (P = 0.01 **)	0.78 (0.76, 0.8) (P = 0 ***)	0.94 (0.92, 0.96) (P = 0 ***)	1.11 (1.09, 1.14) (P = 0 ***)	1.35 (1.32, 1.38) (P = 0 ***)					
	2nd dose	0.98 (0.96, 1) (P = 0.02 **)	0.84 (0.82, 0.87) (P = 0 ***)	0.93 (0.9, 0.96) (P = 0 ***)	1.14 (1.11, 1.17) (P = 0 ***)	1.32 (1.28, 1.36) (P = 0 ***)					
	3rd dose	0.97 (0.92, 1.03) (P = 0.35)	0.89 (0.81, 0.98) (P = 0.02 **)	1.04 (0.96, 1.13) (P = 0.33)	1.09 (1.02, 1.18) (P = 0.02 **)	1.14 (1.06, 1.23) (P = 0 ***)					

*Supplementary Table 6 Adjusted hazard ratio and 95% confidence intervals resulting from the multistate model vaccination transitions for Northern Ireland, Scotland and Wales with a study end date of 31st December 2022. Northern Ireland did not provide results for the ethnicity. The reference for each group is aged 16-17, female, a household occupancy of three, a partially vaccinated household, urban, IMD 2019 quintile of three and white. Asterisk indicates significance of two tailed P values, **P<0.05, ***P<0.01.*

		Sex	Age group		Household n =			Household vaccination status	
Country	Vaccine	Male	12-15	5-11	2	4	5+	Unvaccinated	Fully vaccinated
Wales	1st dose	0.93 (0.92, 0.94) (P = 0 ***)	0.44 (0.44, 0.45) (P = 0 ***)	0.06 (0.06, 0.06) (P = 0 ***)	0.86 (0.83, 0.88) (P = 0 ***)	1.12 (1.11, 1.14) (P = 0 ***)	1.01 (1, 1.03) (P = 0.1)	0.2 (0.19, 0.21) (P = 0 ***)	2.43 (2.39, 2.47) (P = 0 ***)
	2nd dose	0.97 (0.95, 0.98) (P = 0 ***)	0.94 (0.93, 0.95) (P = 0 ***)	0.45 (0.45, 0.46) (P = 0 ***)	0.96 (0.93, 0.99) (P = 0.01 **)	1.04 (1.03, 1.06) (P = 0 ***)	0.93 (0.91, 0.94) (P = 0 ***)	0.66 (0.62, 0.7) (P = 0 ***)	1.28 (1.26, 1.31) (P = 0 ***)
	3rd dose	0.98 (0.96, 1) (P = 0.1)	0.17 (0.16, 0.17) (P = 0 ***)	0 (0, 0.01) (P = 0 ***)	0.92 (0.86, 0.97) (P = 0 ***)	0.97 (0.94, 1) (P = 0.04 **)	0.83 (0.8, 0.86) (P = 0 ***)	0.51 (0.43, 0.6) (P = 0 ***)	1.34 (1.28, 1.39) (P = 0 ***)
Scotland	1st dose	0.94 (0.93, 0.95) (P = 0 ***)	0.54 (0.54, 0.55) (P = 0 ***)	0.12 (0.12, 0.12) (P = 0 ***)	1 (0.98, 1.01) (P = 0.52)	1.04 (1.03, 1.05) (P = 0 ***)	0.84 (0.83, 0.85) (P = 0 ***)	0.34 (0.32, 0.35) (P = 0 ***)	1.47 (1.45, 1.5) (P = 0 ***)
	2nd dose	0.97 (0.96, 0.98) (P = 0 ***)	0.82 (0.81, 0.83) (P = 0 ***)	0.46 (0.45, 0.46) (P = 0 ***)	0.98 (0.96, 1) (P = 0.02 **)	1.03 (1.02, 1.04) (P = 0 ***)	0.94 (0.92, 0.95) (P = 0 ***)	0.91 (0.84, 0.98) (P = 0.02 **)	4 (3.89, 4.12) (P = 0 ***)
	3rd dose	0.91 (0.88, 0.94) (P = 0 ***)	0.13 (0.12, 0.13) (P = 0 ***)	0 (0, 0) (P = 0 ***)	1.04 (0.99, 1.1) (P = 0.14)	0.98 (0.94, 1.02) (P = 0.32)	0.81 (0.78, 0.85) (P = 0 ***)	0.26 (0.1, 0.7) (P = 0.01 **)	1.51 (1.35, 1.69) (P = 0 ***)
Northern Ireland	1st dose	0.88 (0.87, 0.89) (P = 0 ***)	0.43 (0.43, 0.44) (P = 0 ***)	0.02 (0.02, 0.02) (P = 0 ***)	0.82 (0.79, 0.85) (P = 0 ***)	1.15 (1.12, 1.17) (P = 0 ***)	1.11 (1.09, 1.13) (P = 0 ***)	0.24 (0.23, 0.25) (P = 0 ***)	2.28 (2.24, 2.33) (P = 0 ***)
	2nd dose	0.92 (0.91, 0.94) (P = 0 ***)	0.45 (0.45, 0.46) (P = 0 ***)	0.6 (0.58, 0.61) (P = 0 ***)	0.92 (0.88, 0.97) (P = 0 ***)	1.06 (1.03, 1.08) (P = 0 ***)	0.97 (0.94, 0.99) (P = 0.01 **)	0.62 (0.59, 0.67) (P = 0 ***)	1.36 (1.33, 1.39) (P = 0 ***)
	3rd dose	0.98 (0.94, 1.02) (P = 0.3)	0.36 (0.35, 0.38) (P = 0 ***)	0.21 (0.19, 0.23) (P = 0 ***)	0.96 (0.86, 1.07) (P = 0.49)	0.96 (0.91, 1.02) (P = 0.22)	0.91 (0.86, 0.97) (P = 0 ***)	0.65 (0.53, 0.79) (P = 0 ***)	1.28 (1.21, 1.35) (P = 0 ***)

		Urbanicity	IMD 2019 quintile				Ethnicity				
Country	Vaccine	Rural	1 Most	2	4	5 Least	Asian	Mixed	Other	Black	Unknown
Wales	1st dose	0.96 (0.95, 0.97) (P = 0 ***)	0.83 (0.81, 0.84) (P = 0 ***)	0.95 (0.94, 0.97) (P = 0 ***)	1.09 (1.07, 1.11) (P = 0 ***)	1.28 (1.26, 1.31) (P = 0 ***)	1.3 (1.26, 1.33) (P = 0 ***)	0.88 (0.85, 0.91) (P = 0 ***)	0.91 (0.86, 0.96) (P = 0 ***)	0.83 (0.78, 0.88) (P = 0 ***)	0.35 (0.31, 0.4) (P = 0 ***)
	2nd dose	0.93 (0.91, 0.94) (P = 0 ***)	0.91 (0.9, 0.93) (P = 0 ***)	1 (0.98, 1.02) (P = 0.85)	1.05 (1.03, 1.07) (P = 0 ***)	1.17 (1.14, 1.19) (P = 0 ***)	1.16 (1.12, 1.19) (P = 0 ***)	1 (0.96, 1.04) (P = 0.89)	1.03 (0.97, 1.09) (P = 0.4)	0.96 (0.89, 1.03) (P = 0.22)	0.77 (0.66, 0.91) (P = 0 ***)
	3rd dose	0.85 (0.83, 0.88) (P = 0 ***)	0.88 (0.84, 0.91) (P = 0 ***)	0.95 (0.91, 0.99) (P = 0.01 **)	1.01 (0.97, 1.06) (P = 0.48)	1.23 (1.18, 1.27) (P = 0 ***)	1.04 (0.98, 1.11) (P = 0.19)	1.02 (0.93, 1.11) (P = 0.69)	0.81 (0.71, 0.93) (P = 0 ***)	0.75 (0.62, 0.89) (P = 0 ***)	1.3 (0.89, 1.92) (P = 0.18)
Scotland	1st dose	0.99 (0.98, 1) (P = 0.14)	0.72 (0.71, 0.73) (P = 0 ***)	0.85 (0.84, 0.86) (P = 0 ***)	1.12 (1.11, 1.13) (P = 0 ***)	1.32 (1.31, 1.34) (P = 0 ***)	1.32 (1.29, 1.34) (P = 0 ***)	0.93 (0.9, 0.96) (P = 0 ***)	0.89 (0.85, 0.93) (P = 0 ***)	0.83 (0.8, 0.87) (P = 0 ***)	0.94 (0.93, 0.95) (P = 0 ***)
	2nd dose	0.95 (0.94, 0.96) (P = 0 ***)	0.84 (0.83, 0.85) (P = 0 ***)	0.92 (0.91, 0.94) (P = 0 ***)	1.08 (1.07, 1.1) (P = 0 ***)	1.17 (1.15, 1.18) (P = 0 ***)	1.03 (1.01, 1.06) (P = 0.01 **)	0.92 (0.89, 0.96) (P = 0 ***)	0.87 (0.82, 0.93) (P = 0 ***)	0.86 (0.81, 0.91) (P = 0 ***)	1.01 (1, 1.02) (P = 0.17)
	3rd dose	0.81 (0.78, 0.85) (P = 0 ***)	0.84 (0.79, 0.89) (P = 0 ***)	0.88 (0.83, 0.93) (P = 0 ***)	1.09 (1.03, 1.14) (P = 0 ***)	1.26 (1.2, 1.32) (P = 0 ***)	1 (0.92, 1.1) (P = 0.92)	0.93 (0.8, 1.09) (P = 0.37)	0.87 (0.67, 1.12) (P = 0.27)	0.7 (0.54, 0.9) (P = 0.01 **)	0.95 (0.92, 0.99) (P = 0.01 **)
Northern Ireland	1st dose	1.01 (1, 1.03) (P = 0.14)	0.79 (0.77, 0.8) (P = 0 ***)	0.94 (0.92, 0.96) (P = 0 ***)	1.12 (1.09, 1.14) (P = 0 ***)	1.36 (1.34, 1.39) (P = 0 ***)					
	2nd dose	0.96 (0.94, 0.98) (P = 0 ***)	0.84 (0.82, 0.87) (P = 0 ***)	0.92 (0.89, 0.94) (P = 0 ***)	1.15 (1.12, 1.17) (P = 0 ***)	1.35 (1.31, 1.38) (P = 0 ***)					
	3rd dose	0.96 (0.92, 1) (P = 0.06)	0.97 (0.9, 1.05) (P = 0.46)	1.04 (0.97, 1.11) (P = 0.24)	1.14 (1.07, 1.21) (P = 0 ***)	1.21 (1.14, 1.29) (P = 0 ***)					

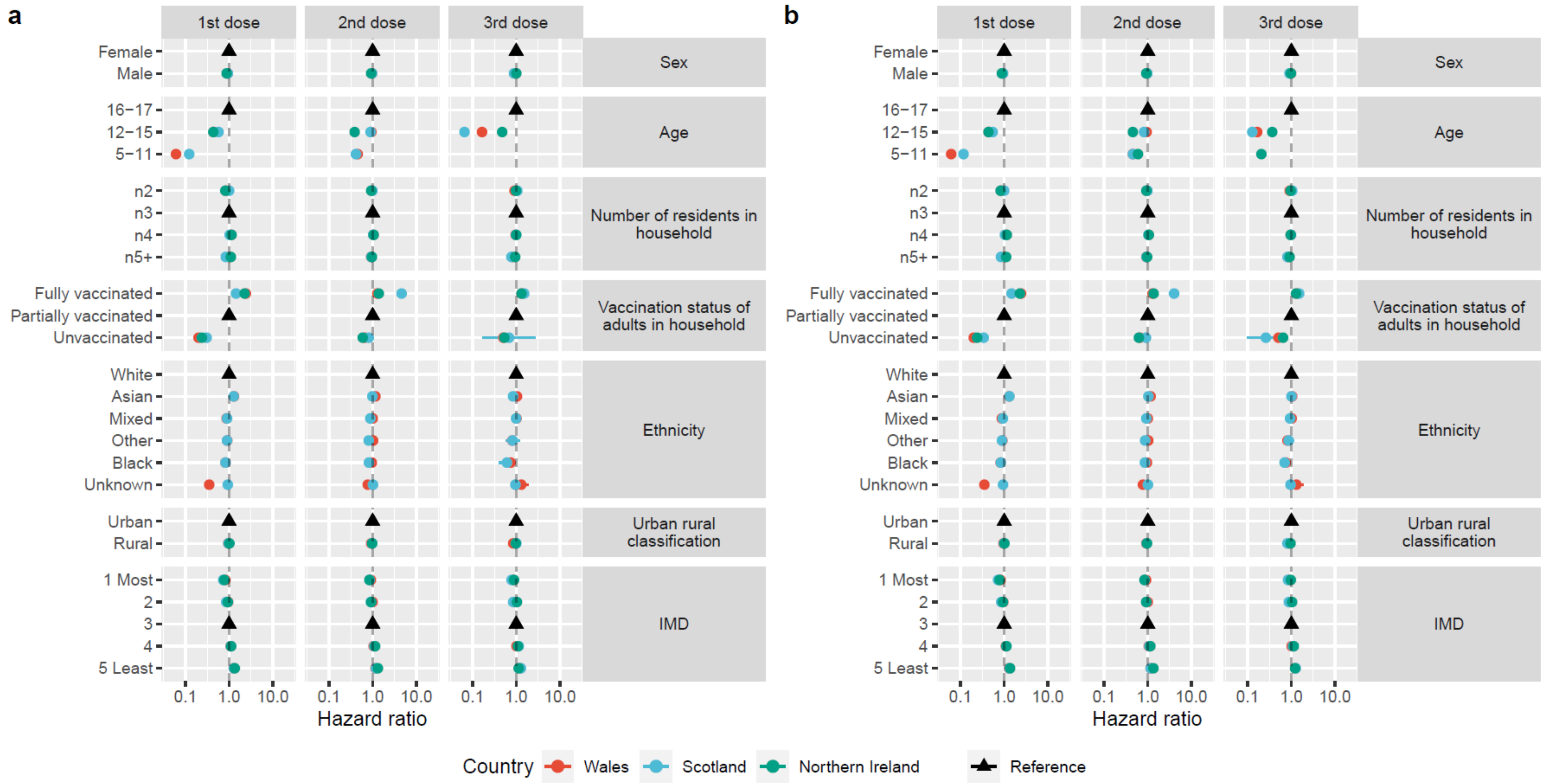

Supplementary Figure 4 Multistate modelling results, adjusted hazard ratio coefficient and 95% confidence intervals resulting from the multistate model for each transition for Northern Ireland, Scotland and Wales (Northern Ireland were not included in the ethnicity analysis) for the a. 4th August 2021 – 31st May 2022 study period and b. the 4th August – 31st December study period. The data used to generate these plots is available in tables 5 (plot a) and 6 (plot b).

Supplementary Table 7 Adjusted hazard ratio and 95% confidence intervals from the meta-analysis for study period ending 31st May 2022. This analysis includes Northern Ireland, Scotland and Wales. Ethnicity results are for Scotland and Wales only.

Variable	*Type*	*Transition*	*aHR*	*95% CI lower*	*95% CI upper*	*Between study variance (τ^2)*
**Age**	*5-11*	1st dose	0.09	0.04	0.17	0.24
		2nd dose	0.44	0.4	0.48	0
	12-15	1st dose	0.48	0.4	0.57	0.02
		2nd dose	0.69	0.39	1.21	0.25
		3rd dose	0.17	0.06	0.53	0.99
**Sex**	*Male*	1st dose	0.92	0.88	0.95	0
		2nd dose	0.95	0.93	0.98	0
		3rd dose	0.96	0.89	1.03	0
**Number of residents in household**	*n2*	1st dose	0.89	0.79	1.01	0.01
		2nd dose	0.96	0.93	0.99	0
		3rd dose	0.98	0.89	1.08	0.01
	n4	1st dose	1.1	1.03	1.17	0
		2nd dose	1.04	1.03	1.05	0
		3rd dose	0.98	0.95	1	0
	n5+	1st dose	0.98	0.84	1.14	0.02
		2nd dose	0.94	0.92	0.96	0
		3rd dose	0.85	0.76	0.95	0.01
**Vaccination status of adults in household**	*Unvaccinated*	1st dose	0.24	0.19	0.31	0.05
		2nd dose	0.68	0.57	0.81	0.02
		3rd dose	0.52	0.45	0.59	0
	Fully vaccinated	1st dose	2	1.45	2.75	0.08
		2nd dose	2.01	0.89	4.53	0.51
		3rd dose	1.34	1.29	1.39	0
**Urban - rural classification**	*Rural*	1st dose	0.98	0.95	1.02	0
		2nd dose	0.96	0.93	0.99	0
		3rd dose	0.94	0.85	1.04	0.01
**IMD 2019 quintile**	*1 Most*	1st dose	0.78	0.73	0.83	0
		2nd dose	0.87	0.83	0.91	0
		3rd dose	0.85	0.79	0.92	0
	2	1st dose	0.92	0.86	0.97	0
		2nd dose	0.94	0.89	1	0
		3rd dose	0.94	0.85	1.05	0.01
	4	1st dose	1.1	1.09	1.11	0
		2nd dose	1.09	1.04	1.14	0
		3rd dose	1.08	1	1.16	0
	5 Least	1st dose	1.31	1.27	1.35	0
		2nd dose	1.22	1.13	1.32	0
		3rd dose	1.22	1.15	1.29	0
**Ethnicity**	*Asian*	1st dose	1.28	1.26	1.3	0
		2nd dose	1.07	0.91	1.25	0.01
		3rd dose	0.95	0.78	1.16	0.02
	Mixed	1st dose	0.89	0.87	0.91	0
		2nd dose	0.94	0.83	1.07	0.01
		3rd dose	1.01	0.94	1.1	0
	Other	1st dose	0.9	0.87	0.94	0
		2nd dose	0.91	0.73	1.15	0.03
		3rd dose	0.82	0.72	0.92	0
	Black	1st dose	0.83	0.8	0.86	0
		2nd dose	0.89	0.76	1.03	0.01
		3rd dose	0.72	0.61	0.85	0
	Unknown	1st dose	0.57	0.22	1.5	0.48
		2nd dose	0.9	0.69	1.18	0.03
		3rd dose	1.05	0.8	1.39	0.03

Supplementary Table 8 Adjusted hazard ratio and 95% confidence intervals from the meta-analysis for study period ending 31st December 2022. This analysis includes Northern Ireland, Scotland and Wales. Ethnicity results are for Scotland and Wales only.

**Variable**	*Type*	*Transition*	*aHR*	*95% CI lower*	*95% CI upper*	*Between study variance (τ^2)*
**Age**	*5-11*	1st dose	0.05	0.02	0.14	0.74
		2nd dose	0.5	0.42	0.59	0.02
		3rd dose	0.47	0.41	0.54	0.01
	12-15	1st dose	0.7	0.46	1.09	0.15
		2nd dose	0.2	0.11	0.37	0.3
		3rd dose	0.92	0.88	0.95	0
**Sex**	*Male*	1st dose	0.95	0.92	0.98	0
		2nd dose	0.95	0.91	1	0
		3rd dose	0.89	0.79	1	0.01
**Number of residents in household**	*n2*	1st dose	0.96	0.93	0.99	0
		2nd dose	0.97	0.9	1.06	0
		3rd dose	1.1	1.04	1.17	0
	n4	1st dose	1.04	1.03	1.05	0
		2nd dose	0.97	0.95	0.99	0
		3rd dose	0.98	0.84	1.15	0.02
	n5+	1st dose	0.94	0.92	0.96	0
		2nd dose	0.85	0.79	0.91	0
		3rd dose	0.25	0.19	0.34	0.07
**Vaccination status of adults in household**	*Unvaccinated*	1st dose	0.72	0.57	0.9	0.04
		2nd dose	0.54	0.43	0.7	0.02
		3rd dose	2.01	1.48	2.74	0.07
	Fully vaccinated	1st dose	1.91	0.93	3.94	0.41
		2nd dose	1.35	1.25	1.47	0
		3rd dose	0.78	0.72	0.84	0
**Urban - rural classification**	*Rural*	1st dose	0.87	0.82	0.91	0
		2nd dose	0.89	0.82	0.97	0
		3rd dose	0.91	0.85	0.98	0
**IMD 2019 quintile**	*1 Most*	1st dose	0.95	0.9	1	0
		2nd dose	0.95	0.87	1.05	0.01
		3rd dose	1.11	1.09	1.13	0
	2	1st dose	1.09	1.04	1.15	0
		2nd dose	1.07	1.01	1.15	0
		3rd dose	1.32	1.28	1.37	0
	4	1st dose	1.22	1.11	1.34	0.01
		2nd dose	1.23	1.2	1.27	0
		3rd dose	0.99	0.96	1.02	0
	5 Least	1st dose	0.94	0.93	0.96	0
		2nd dose	0.87	0.79	0.96	0.01
		3rd dose	1.31	1.29	1.33	0
**Ethnicity**	*Asian*	1st dose	1.09	0.98	1.22	0.01
		2nd dose	1.03	0.98	1.08	0
		3rd dose	0.9	0.85	0.96	0
	Mixed	1st dose	0.96	0.89	1.04	0
		2nd dose	1	0.92	1.07	0
		3rd dose	0.9	0.87	0.93	0
	Other	1st dose	0.94	0.8	1.11	0.01
		2nd dose	0.82	0.73	0.93	0
		3rd dose	0.83	0.8	0.86	0
	Black	1st dose	0.9	0.81	1.01	0
		2nd dose	0.73	0.63	0.85	0
		3rd dose	0.58	0.22	1.51	0.48
	Unknown	1st dose	0.89	0.69	1.16	0.03
		2nd dose	1.05	0.79	1.39	0.03
		3rd dose	0.05	0.02	0.14	0.74

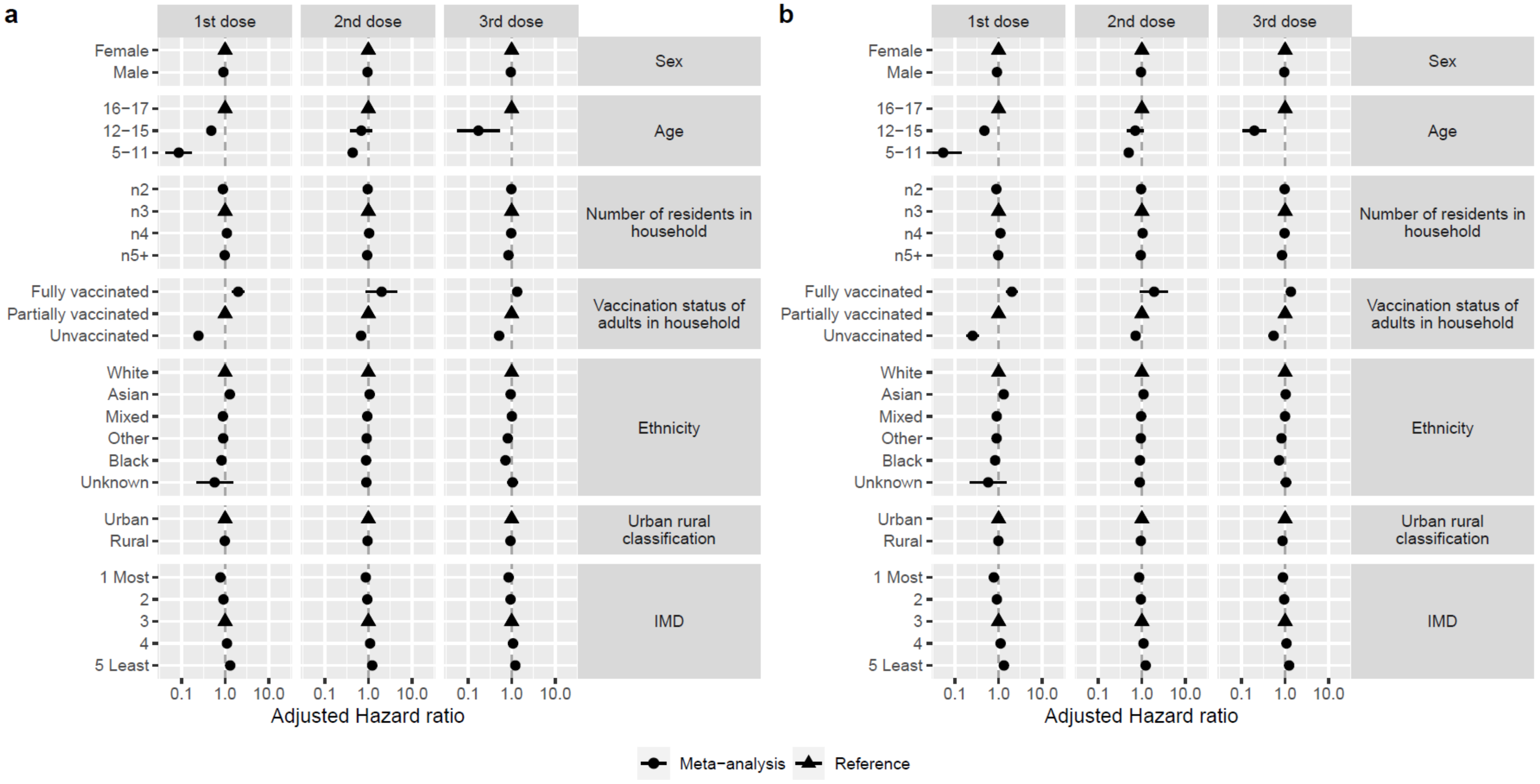

Supplementary Figure 5 Meta-analysis results, adjusted hazard ratio coefficients and 95% confidence intervals produced from the random effects model for Northern Ireland, Scotland and Wales (Northern Ireland were not included in the ethnicity analysis) for a. the 4th August 2021 – 31st May 2022 study period and b. the 4th August – 31st December study period. The data used to generate these plots is available in tables 7 (plot a) and 8 (plot b).

Cohort selection

All cohorts generated by each nation were subjected to the following parameters to clean the data.

- In cohort between July 2021 and May 2022 (this allows us to determine if they were infected up to 28 days prior to the start of the study).
- Has week of birth and sex recorded
- Is aged between 5 and 17 between Aug 2021 and May 2022
- Is resident in country of analysis from Jan 2020
- Has unique residential ID
- Household less than 10
- Minimum of 1 adult in household
- Registered with GP accessible to the countries data linkage.
- Does not have a hospital spell of more than 1 week during study period
- Has good vaccine records (UK vaccination, valid vaccine name and date, before today, valid dose sequence, minimum 28 days between vaccinations)
- If CYP has 1st and 2nd dose then with ChAdOx1 adenoviral (Oxford-AstraZeneca), mRNA-1273 (Moderna) or BNT162b2 mRNA (PfizerBioNTech)
- If CYP has booster then with Oxford-AstraZeneca, Moderna or PfizerBioNTech
- Date of dose 1 is after age group eligibility
