# Supplementary material for: Uptake of COVID-19 vaccinations amongst 3,433,483 children and young people: meta-analysis of UK prospective cohorts
Source: Nat Commun. 2024 Mar 15;15:2363. doi: 10.1038/s41467-024-46451-0 (PMC10943015; doi:10.1038/s41467-024-46451-0)
Supplement: Supplementary file 3 — Reporting Summary [file 41467_2024_46451_MOESM3_ESM.pdf]

nature portfolio

Corresponding author(s): Aldridge S. J.

Last updated by author(s): Jun 9, 2023

# Reporting Summary

Nature Portfolio wishes to improve the reproducibility of the work that we publish. This form provides structure for consistency and transparency in reporting. For further information on Nature Portfolio policies, see our Editorial Policies and the Editorial Policy Checklist.

## Statistics

For all statistical analyses, confirm that the following items are present in the figure legend, table legend, main text, or Methods section.

| n/a | Confirmed | |
|---|---|---|
| ☐ | ☒ | The exact sample size (*n*) for each experimental group/condition, given as a discrete number and unit of measurement |
| ☒ | ☐ | A statement on whether measurements were taken from distinct samples or whether the same sample was measured repeatedly |
| ☐ | ☒ | The statistical test(s) used AND whether they are one- or two-sided<br>*Only common tests should be described solely by name; describe more complex techniques in the Methods section.* |
| ☐ | ☒ | A description of all covariates tested |
| ☐ | ☒ | A description of any assumptions or corrections, such as tests of normality and adjustment for multiple comparisons |
| ☐ | ☒ | A full description of the statistical parameters including central tendency (e.g. means) or other basic estimates (e.g. regression coefficient) AND variation (e.g. standard deviation) or associated estimates of uncertainty (e.g. confidence intervals) |
| ☐ | ☒ | For null hypothesis testing, the test statistic (e.g. *F*, *t*, *r*) with confidence intervals, effect sizes, degrees of freedom and *P* value noted<br>*Give P values as exact values whenever suitable.* |
| ☒ | ☐ | For Bayesian analysis, information on the choice of priors and Markov chain Monte Carlo settings |
| ☒ | ☐ | For hierarchical and complex designs, identification of the appropriate level for tests and full reporting of outcomes |
| ☒ | ☐ | Estimates of effect sizes (e.g. Cohen's *d*, Pearson's *r*), indicating how they were calculated |

*Our web collection on statistics for biologists contains articles on many of the points above.*

## Software and code

Policy information about availability of computer code

| | |
|---|---|
| Data collection | This works makes use of pseudonymised health and administrative data held within the trusted research environments for each UK nation. |
| Data analysis | National level data analysis scripts were created using Rstudio v4.1.3 for running within the TREs. Meta-analsysis scripts were created and performed using Rstudio v4.2.0. Analystical R scripts made us of using the Survival, Mstate and Metafor packages. All codes used are publicly available at https://github.com/HDRUK/DaCVaP/tree/main/DaCVaP2/Children-and-Young-People. |

For manuscripts utilizing custom algorithms or software that are central to the research but not yet described in published literature, software must be made available to editors and reviewers. We strongly encourage code deposition in a community repository (e.g. GitHub). See the Nature Portfolio guidelines for submitting code & software for further information.

## Data

Policy information about availability of data

All manuscripts must include a data availability statement. This statement should provide the following information, where applicable:

- Accession codes, unique identifiers, or web links for publicly available datasets
- A description of any restrictions on data availability
- For clinical datasets or third party data, please ensure that the statement adheres to our policy

This works makes use of pseudonymised health and administrative data held within the trusted research environments for each UK nation. Aggregate data on COVID-19 vaccinations of CYP in the UK are provided in this publication's supplementary material. The patient-level data underlying this article provided by the

RCGP and RSC database, NHAIS, EAVE II platform and the SAIL Databank cannot be shared publicly due to data protection and confidentiality requirements. Data can be made available to approved researchers for analysis after securing relevant permissions from the data holders via the relevant approval pathways.

## Research involving human participants, their data, or biological material

Policy information about studies with human participants or human data. See also policy information about sex, gender (identity/presentation), and sexual orientation and race, ethnicity and racism.

| | |
|---|---|
| Reporting on sex and gender | We refer to sex throughout the paper, and include this as a covariate in analysis. Sex is taken from a combination of primary care and administrative sources, and will likely represent a combination of sex and gender depending on the method of obtaining. We restrict the cohorts to only include participants who have a male or female sex assigned to their data. |
| Reporting on race, ethnicity, or other socially relevant groupings | We did not include social groupings in out main analysis, however it was included as a sensitivity analysis in the supplementary. We found that ethnicity and deprivation were both statistically significant variables, however when included in the multivariate analysis they did not impact on other variables.<br>Ethnicity information was taken from a combination of health and administrative sources to classify individuals into categories. Deprivation was scored using an indicator of Multiple Deprivation (IMD), also drawing data from a combination of health and administrative sources. |
| Population characteristics | We included several population characteristics collected at the individual level. These include, including age (5-11 years old, 12-15 years old, or 16-17 years old), sex (male or female), household size (2-5+) and household vaccination status (unvaccinated, partially vaccinated, or fully vaccinated). |
| Recruitment | Electronic health records are collected as part of a patients' routine healthcare. |
| Ethics oversight | In England, ethical approval was granted by the Health Research Authority London Central Research Ethics Committee (reference number REC reference 21/HRA/2786; integrated research application system number 30174). In Northern Ireland, study approval was granted by the Honest Broker Service (HBS) Governance Board (project number 064; the HBS process does not require separate National Research Ethics Service governance approval). In Scotland, ethical approval was granted by the National Research Ethics Service Committee (Southeast Scotland 02; reference number 12/SS/0201), and the approval for data linkage was granted by the Public Benefit and Privacy Panel for Health and Social Care (reference number 1920–0279). In Wales, research conducted within the Secure Anonymised Information Linkage Databank was done with the permission and approval of the independent Information Governance Review Panel (project number 0911). Individual written patient consent was not required for this study. |

Note that full information on the approval of the study protocol must also be provided in the manuscript.

# Field-specific reporting

Please select the one below that is the best fit for your research. If you are not sure, read the appropriate sections before making your selection.

☒ Life sciences ☐ Behavioural & social sciences ☐ Ecological, evolutionary & environmental sciences

For a reference copy of the document with all sections, see nature.com/documents/nr-reporting-summary-flat.pdf

# Life sciences study design

All studies must disclose on these points even when the disclosure is negative.

| | |
|---|---|
| Sample size | Sample size was the whole eligible population of 5-17 year olds available for England, Northern Ireland, Scotland and Wales. |
| Data exclusions | Participants who did not meet the following criteria were excluded: Lived in UK from Jan 2020, and were living in a UK region between 7th July 2021 and 31st May 2022, aged from 5 to 17 with week of birth recorded, registered with a GP accessible to the countries data linkage, recorded as male or female, a unique ID, living in a household of less than 10 with a minimum of 1 adult present, and no hospital stay of more than 1 week during the study period. Participants were also excluded depending on the vaccine information available. Participants who did not meet the following vaccination requirements were excluded: UK vaccination with a valid vaccine name date and dose sequence with a minimum of 28 days between vaccines, the vaccines must be ChAdOx1 adenoviral (Oxford-AstraZeneca), mRNA-1273 (Moderna) or BNT162b2 mRNA (PfizerBioNTech), and must be administered after eligibility for their age group. |
| Replication | Code available for replication |
| Randomization | This was an observational study and randomisation was not possible |
| Blinding | This was an observational study and blinding was not possible |

# Reporting for specific materials, systems and methods

We require information from authors about some types of materials, experimental systems and methods used in many studies. Here, indicate whether each material, system or method listed is relevant to your study. If you are not sure if a list item applies to your research, read the appropriate section before selecting a response.

## Materials & experimental systems

| n/a | Involved in the study |
| --- | --- |
| ☒ | ☐ Antibodies |
| ☒ | ☐ Eukaryotic cell lines |
| ☒ | ☐ Palaeontology and archaeology |
| ☒ | ☐ Animals and other organisms |
| ☒ | ☐ Clinical data |
| ☒ | ☐ Dual use research of concern |
| ☒ | ☐ Plants |

## Methods

| n/a | Involved in the study |
| --- | --- |
| ☒ | ☐ ChIP-seq |
| ☒ | ☐ Flow cytometry |
| ☒ | ☐ MRI-based neuroimaging |
